# Cyclophilin D Regulates the Nuclear Translocation of AIF, Cardiac Endothelial Cell Necroptosis and Murine Cardiac Transplant Injury

**DOI:** 10.3390/ijms222011038

**Published:** 2021-10-13

**Authors:** Adnan Qamar, Jianqi Zhao, Laura Xu, Patrick McLeod, Xuyan Huang, Jifu Jiang, Weihua Liu, Aaron Haig, Zhu-Xu Zhang

**Affiliations:** 1 Matthew Mailing Centre for Translational Transplantation Studies, London Health Sciences Centre, B4-231, 339 Windermere Road, London, ON N6A 5A5, Canada; aqamar1@tuftsmedicalcenter.org (A.Q.); jzhao446@uwo.ca (J.Z.); lxu332@uwo.ca (L.X.); Patrick.Mcleod@lhsc.on.ca (P.M.); Xuyan.Huang@lhsc.on.ca (X.H.); Jifu.Jiang@lhsc.on.ca (J.J.); 2 Department of Pathology, Western University, 1151 Richmond Street, London, ON N6A 3K7, Canada; weihua.liu@schulich.uwo.ca (W.L.); Aaron.Haig@lhsc.on.ca (A.H.); 3 Department of Rheumatology and Immunology, The First Hospital of Jilin University, 3808 Jiefang Road, Changchun 130021, China; 4 Multi-Organ Transplant Program, London Health Sciences Centre, London, ON N6A 5A5, Canada; 5 Division of Nephrology, Department of Medicine, Western University, London, ON N6A 3K7, Canada

**Keywords:** hypoxia, ischemia, necroptosis, CypD, AIF, endothelial cell, cardiac, transplantation

## Abstract

Ischemia-reperfusion injury (IRI) is an inevitable consequence of organ transplant procedure and associated with acute and chronic organ rejection in transplantation. IRI leads to various forms of programmed cell death, which worsens tissue damage and accelerates transplant rejection. We recently demonstrated that necroptosis participates in murine cardiac microvascular endothelial cell (MVEC) death and murine cardiac transplant rejection. However, MVEC death under a more complex IRI model has not been studied. In this study, we found that simulating IRI conditions in vitro by hypoxia, reoxygenation and treatment with inflammatory cytokines induced necroptosis in MVECs. Interestingly, the apoptosis-inducing factor (AIF) translocated to the nucleus during MVEC necroptosis, which is regulated by the mitochondrial permeability molecule cyclophilin D (CypD). Furthermore, CypD deficiency in donor cardiac grafts inhibited AIF translocation and mitigated graft IRI and rejection (*n* = 7; *p* = 0.002). Our studies indicate that CypD and AIF play significant roles in MVEC necroptosis and cardiac transplant rejection following IRI. Targeting CypD and its downstream AIF may be a plausible approach to inhibit IRI-caused cardiac damage and improve transplant survival.

## 1. Introduction

Ischemic heart disease remains a top health problem worldwide and affects around 100 million individuals. The current prevalence rate is above 1600 per 100,000 people globally, with higher rates in Western countries (~3000) and lower in South Asian countries (~1000) [1,2]. When heart disease progresses to severe heart failure, transplantation may be the only remaining option. Ischemia-reperfusion injury (IRI) is an inevitable consequence of an organ transplant procedure, as all donor organs, including the heart, kidney, liver and lung, have to be stored and transported before being transplanted into the recipients. IRI leads to primary graft dysfunction and also has deleterious long-term effects on graft survival. Unfortunately, premature graft failure has emerged as one of our greatest transplant challenges, as 50% of all heart transplants fail over time. In the clinic, ischemic time correlates with delayed heart graft function and organ failure, despite the development of novel and effective immunosuppressive agents that have significantly reduced the rejection rates [3,4,5,6]. Amongst the early complications, primary graft dysfunction remains the leading cause of mortality in heart transplant recipients, with a 30-day mortality rate of ~30% [7]. The cold storage of heart grafts is currently limited to approximately 6 h in clinical transplantation [3,4,5,6]. Oxidative stress, enzymatic activity or inflammatory responses have been considered as therapeutic targets of IRI [8,9,10,11]. In transplantation, IRI can trigger a nonspecific inflammatory response that contributes to the immunogenicity of the allograft and adversely affects allograft survival and function. IRI contributes to alloimmune injury and influences short-term, as well as long-term, allograft survival and function [6,12]. Currently, there are no specific treatments available to prevent IRI [3,4]. Novel therapeutic strategies are required to preserve heart function and improve clinical outcomes for patients. 

The endothelium is the first barrier for graft injury and can produce various pro-inflammatory cytokines [13,14], as well as permeability for the recruitment of leukocytes, which worsens graft injury [15,16]. Endothelial cells play a crucial role in the inflammatory response associated with IRI. Endothelial cells undergo many of the pathological changes associated with IRI, including the disruption of critical energy-dependent cellular systems, intracellular acidosis, electrolyte imbalances, cellular swelling and cell death, all of which are potential therapeutic targets [17,18,19,20]. The endothelium may be more vulnerable than smooth muscle cells and cardiomyocytes during IRI [21,22,23,24]. However, most studies that focus on myocytes and endothelial cells have not yet been well studied. 

The cumulative pathological changes induced by IRI may lead to cell death and worsen organ injury and accelerate transplant rejection [25,26]. Several different forms of programmed cell death are now known, broadly classified as apoptosis and regulated necrosis types. The latter includes many further types, including necroptosis, pyroptosis, and ferroptosis, among others. In our previous studies, we have demonstrated that necroptosis participates in mouse cardiac microvascular endothelial cell (MVEC) death and plays an important role in murine cardiac and renal transplant rejection [27,28,29,30,31]. However, MVEC death under more complex IRI models has not been studied. 

In this study, we demonstrate that necroptosis plays a significant role in hypoxia-reoxygenation-induced MVEC death. We have found that cyclophilin D (CypD) and apoptosis-induced factor (AIF) are the downstream effectors of necroptosis. CypD regulates the nuclear translocation of AIF. CypD deficiency in donor cardiac grafts inhibits AIF translocation and attenuates transplant IRI and rejection.

## 2. Results

### 2.1. In Vitro Simulation of Ischemia and Reperfusion Promotes Necroptosis in MVECs

We have previously found that necroptosis plays an important role in MVEC death and heart graft rejection [27,28]. However, the role of necroptosis in a clinically relevant IRI model have not yet been studied. We wanted to establish an in vitro condition to simulate in vivo IRI in murine cardiac transplantation.

**Figure 1:**
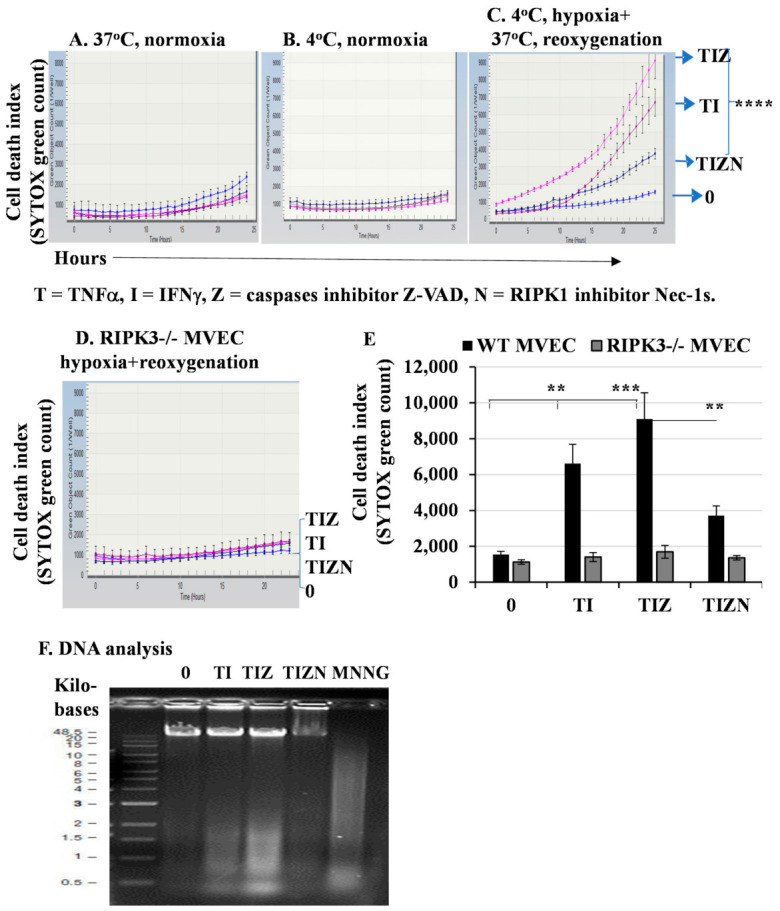
In vitro simulating IRI promotes necroptosis in MVECs. MVECs from wild-type (WT) B6 mice (**A**–**C**) or RIPK3^−/−^ mice (**D**) were seeded in a 96-well plate in triplicate. The cell culture plate was subjected to hypoxia for 24 h in anaerobic GENbags at 4 °C followed by normal cell culture conditions at 37 °C with 20% O_2_ and 5% CO_2_. TNFα (T) at 50 ng/mL, IFNγ (I) at 50 ng/mL, z-VAD-fmk (Z) at 30 μM and Nec-1s (N) at 10 μM were added. SYTOX green at 100 nM was used as a nuclear counterstain to indicate dead cells. Cell death was detected and quantified in the IncuCyte Live Cell Image System. (**E**) Data at 24 h post-reoxygenation are shown as the mean ± standard deviation (SD) and represent at least 3 independent experiments. ** *p* ≤ 0.01, *** *p* ≤ 0.001 and **** *p* ≤ 0.0001, 1-way ANOVA and Tukey’s multiple comparisons test. (**F**) DNA degradation analysis. MVECs were seeded in 6-well plates and subjected to the in vitro IRI treatment as above. DNA was isolated, and an equal amount of DNA (2 μg) was loaded onto 0.8% agarose gel for electrophoresis. MNNG (15 μM) was used as a positive control to induce DNA degradation. The same experiment was repeated three times, and similar results were obtained.

TNFα and IFNγ are hallmarks of inflammation in IRI and, thus, were used together in our study. They induced a minimal level of MVEC death after treatment under normoxic culture conditions at 37 °C and 4 °C (Figure 1A,B). To simulate the in vivo organ ischemia-reperfusion conditions, MVECs were subjected to in vitro 24-h cold hypoxia, followed by a warm reoxygenation treatment. The treatment of cold hypoxia and warm reoxygenation alone only induced minimal levels of cell death (Figure 1C,E). However, cold hypoxia and warm reoxygenation treatments with the addition of TNFα and IFNγ induced a significant level of cell death (Figure 1C,E). Next, we studied the mechanism of cell death. It is well-established that IRI induces apoptosis, which is mainly mediated by a caspase cascade. However, the addition of pan-caspase inhibitor z-VAD-fmk did not prevent cell death, as shown in Figure 1C,E. z-VAD-fmk is a pan-caspase inhibitor and prevents apoptosis; therefore, cell death following treatment with z-VAD-fmk was suggestive of necroptosis. Indeed, the addition of the receptor-interacting protein kinase-1 (RIPK1) inhibitor necrostatin-1s (Nec-1s) significantly inhibited cell death (Figure 1C,E). To further confirm necroptosis, we used MVECs developed from RIPK3-deficient mice [28]. Interestingly, cell death was prevented in RIPK3^−/−^ MVEC (Figure 1D,E). 

A hallmark of cell death is DNA fragmentation. DNA fragmentation in necroptosis has not been well-studied, while apoptosis-induced DNA fragmentation via caspases is well-characterized. Next, we wanted to detect DNA degradation by electrophoresis. The treatment of hypoxia and reoxygenation together with TNFα and IFNγ induced a significant level of DNA fragmentation that could not be inhibited by caspase inhibitor z-VAD-fmk (Figure 1F). However, the addition of RIPK1 inhibitor Nec-1 significantly inhibited DNA fragmentation (Figure 1F), suggesting necroptosis-induced DNA damage. Taken together, our data indicate that the in vitro simulation of IRI conditions promote necroptosis and DNA degradation in MVECs. Thus, we investigated the downstream mechanism of necroptosis.

### 2.2. CypD, Not ROS, Contributes to MVECs Necroptosis under In Vitro Simulating IRI Condition

**Figure 2:**
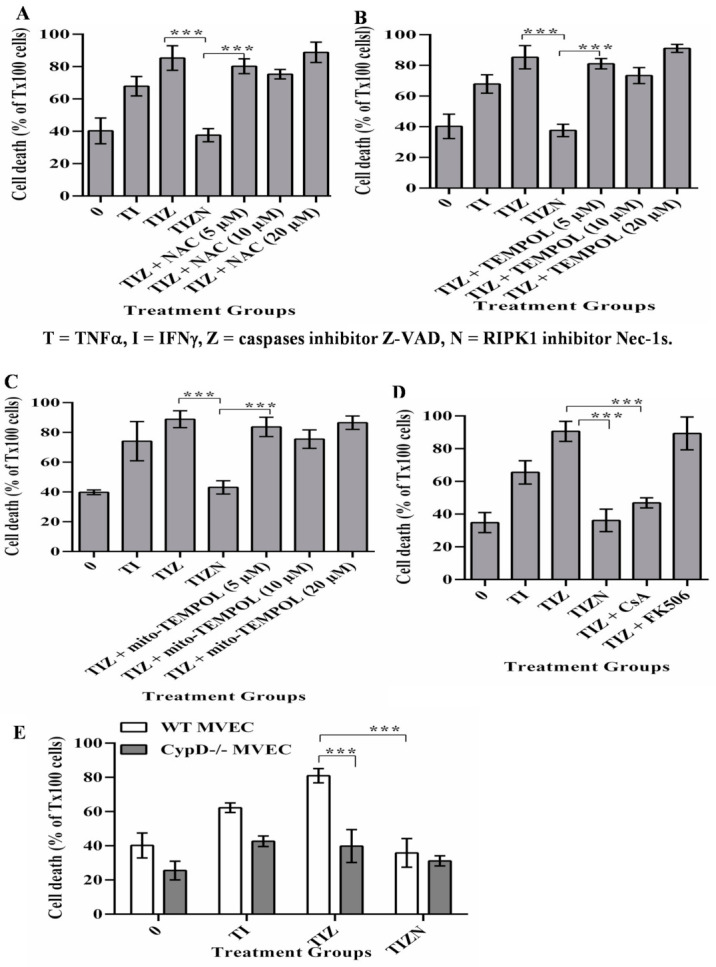
CypD, not ROS, contributes to MVEC necroptosis under the in vitro simulation of IRI conditions. (**A**–**C**). MVECs were subjected to cold hypoxia-reoxygenation with cytokines, as described in Figure 1. ROS scavenger NAC, TEMPOL or Mito-TEMPOL were added, respectively. A small amount of (0.1%) Triton X-100 (Tx100) was used to induce maximum death with 100% cell staining of SYTOX green. % EC death = sample count/count of Triton-X100-treated cells. Data are shown at 24 h post-reoxygenation as the mean ± SD of triplicates and representative of at least 3 independent experiments. (**D**) CypD inhibitor—CsA and control FK506 at 10 μM was added before hypoxia. Data are shown as the mean ± SD and representative of at least 3 independent experiments. (**E**) Wild-type (WT) and CypD^−/−^ MVECs were subjected to in vitro cold hypoxia-reoxygenation with cytokines, as in Figure 1. Data are shown as the mean ± SD and representative of at least 3 independent experiments. *** *p* ≤ 0.001; 1-way ANOVA and Tukey’s multiple comparisons test.

It has well-established that mitochondria play a crucial role for ischemia/reperfusion-induced cell death and organ injury. The dysfunction of mitochondria leads to the incomplete reduction of oxygen and also provides a link to reactive oxygen species (ROS) generation, which leads to cellular damage and is a strong trigger for apoptosis and necroptosis [26,32]. The role of ROS-mediated cellular injury in inducing apoptosis in the setting of IRI has been extensively studied [33,34]. Furthermore, the role of ROS in inducing necroptosis has been studied in models of IRI [25,26,35,36,37]. However, the direct role of mitochondria in programmed necrosis remains controversial [32,38,39,40,41]. To determine the role of ROS in MVEC necroptosis under the in vitro simulation of IRI conditions, the ROS scavengers, tetramethylpiperidine-oxyl (TEMPOL) and N-acetyl-L-cysteine (NAC) were added into the culture. Interestingly, the addition of TEMPOL or NAC did not reverse necroptosis (Figure 2A,B). The addition of the mitochondrion-specific ROS scavenger mito-TEMPOL also did not reverse necroptosis (Figure 2C). This finding ruled out the role of ROS in necroptosis in our model. 

Mitochondrial dysfunction leads to increasing their membrane permeability, which is largely regulated by CypD [42,43]. Studies investigating the role of CypD-mediated mitochondrial membrane permeability in necroptosis have yielded contradictory results [29,32,38,39,40,41,42,44]. Cyclosporine A (CsA) is a classical immunosuppressive drug in clinical transplantation and is a potent CypD inhibitor. In our study, the addition of CsA reversed necroptosis in MVECs (Figure 2D). Besides blocking CypD, CsA is a calcineurin inhibitor and a potent immunosuppressive drug. To study the participation of calcineurin in necroptosis, the non-CypD-binding calcineurin inhibitor FK-506 was used. However, the addition of FK-506 did not inhibit necroptosis (Figure 2D), confirming that CypD-mediated mitochondrial membrane permeability participates in necroptosis. To further confirm the role of CypD in necroptosis, MVECs developed from CypD^−/−^ mice were subjected to an in vitro simulating IRI treatment. As with CypD inhibition by CsA, CypD^−/−^ MVECs resisted necroptosis (Figure 2E), confirming the CypD contribution to necroptosis in MVECs.

### 2.3. AIF Translocates to the Nucleus during Necroptosis under In Vitro Simulation of IRI Conditions

CypD activity increases the mitochondrial membrane permeability, resulting the release of mitochondrial molecules [42]. Previous studies showed that AIF translocates from the mitochondrion to the nucleus and induces caspase-independent cell death [45,46,47]. Next, we investigated the possibility that CypD activity results in the nuclear translocation of AIF and mediates necroptosis. 

**Figure 3:**
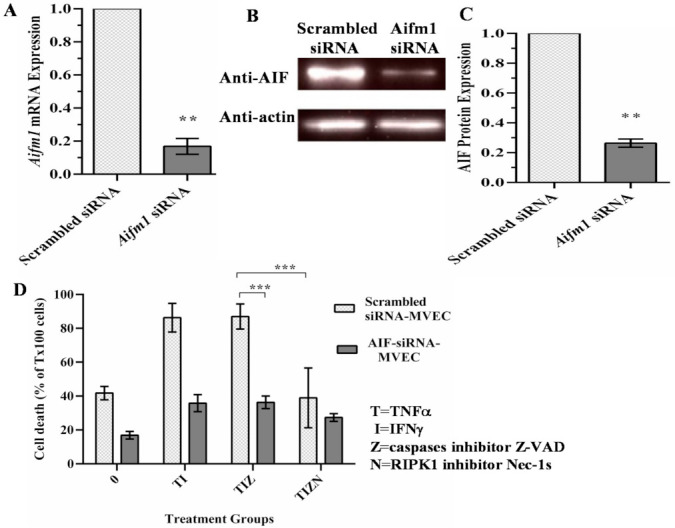
AIF silencing prevents necroptosis in endothelial cells. (**A**) siRNA-induced silencing of *AIF* was confirmed by qPCR. *β-actin* was used as an endogenous control for the gene expression analysis. Data at 48 h post-transfection are shown as the mean ± SD and representative of 3 independent qPCR experiments. *n* = 3; ** *p* ≤ 0.01; Student’s *t*-test. (**B**) The reduction in the protein expression of AIF-silenced cells was confirmed by a Western blot analysis at 72 h post-transfection. β-actin was used as a loading control. (**C**) The reduction in the level of AIF protein expression was measured in 3 independent experiments. *n* = 3; ** *p* ≤ 0.01; Student’s *t*-test. (**D**) Scrambled siRNA-transfected and AIF-silenced MVECs were harvested 48 h post-transfection and subjected to the in vitro simulating IRI treatment, as in Figure 1. Data are shown as the mean ± SD and representative of 3 independent experiments. *** *p* ≤ 0.001; 2-way ANOVA and Tukey’s multiple comparisons test.

To study the role of AIF in necroptosis, AIF expression was silenced in MVECs using siRNA. AIF silencing was confirmed by PCR (Figure 3A) and Western blot (Figure 3B,C). AIF-silenced endothelial cells were subjected to the in vitro simulation of IRI treatment. Compared to scrambled siRNA-transfected cells, AIF-silenced cells showed a significant reduction in cell death (Figure 3D). This finding indicates that AIF has a critical role in the necroptotic pathway.

**Figure 4:**
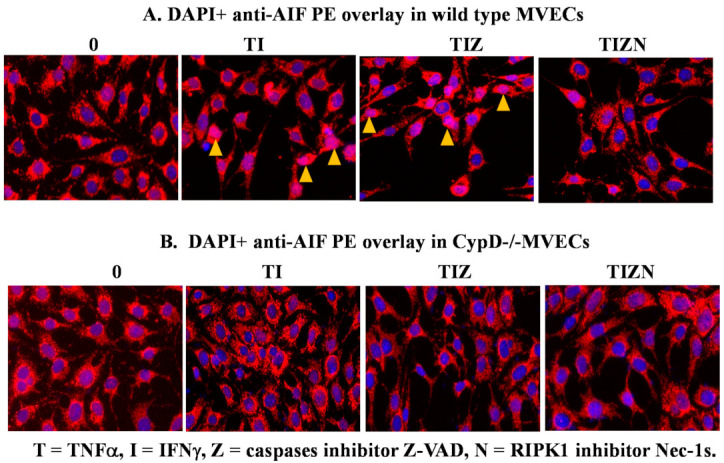
AIF translocation to the nucleus in necroptotic MVECs. Wild-type B6 MVECs (**A**) and CypD^−/−^ MVECs (**B**) were subjected to the in vitro IRI treatment, as above. The MVECs were fixed with 4% formaldehyde solution after 24 h of reoxygenation and permeabilized with 0.1% Triton X-100. The cells were incubated with antimurine AIF antibody, followed by anti-rabbit PE-conjugated antibody. The nucleus was stained with 300-nM DAPI. Nuclear AIF staining (pink color) is pointed to by arrows. Images were captured under 200 times magnification under fluorescent microscopy. The same experiment was repeated at least 3 times. Representative cell images are shown.

Previous studies have demonstrated that, following its release into the cytosol from the mitochondria, AIF translocates to the nucleus and participates in DNA degradation [45,46,47]. We examined if this is the case in our study by immunocytochemistry. In the control, AIF stays in the cytoplasm (Figure 4A). However, AIF translocation to the nucleus is observed in the simulation of IRI conditions (Figure 4A). Adding pan-caspase inhibitor z-VAD-fmk did not prevent AIF translocation (Figure 4A). However, the addition of the RIPK1 inhibitor Nec-1s prevented AIF translocation (Figure 4A), suggesting the role of AIF translocation during necroptosis. 

In Figure 2, we showed that CypD participates in necroptosis and CypD deletion, or inhibition prevents MVEC necroptosis. Furthermore, we studied whether CypD regulates AIF translocation. Interestingly, AIF translocation was not observed in CypD-deficient MVECs under the necroptosis induction conditions in vitro (Figure 4B). These data indicate that CypD participates in the nuclear translocation of AIF during MVEC necroptosis.

**Figure 5:**
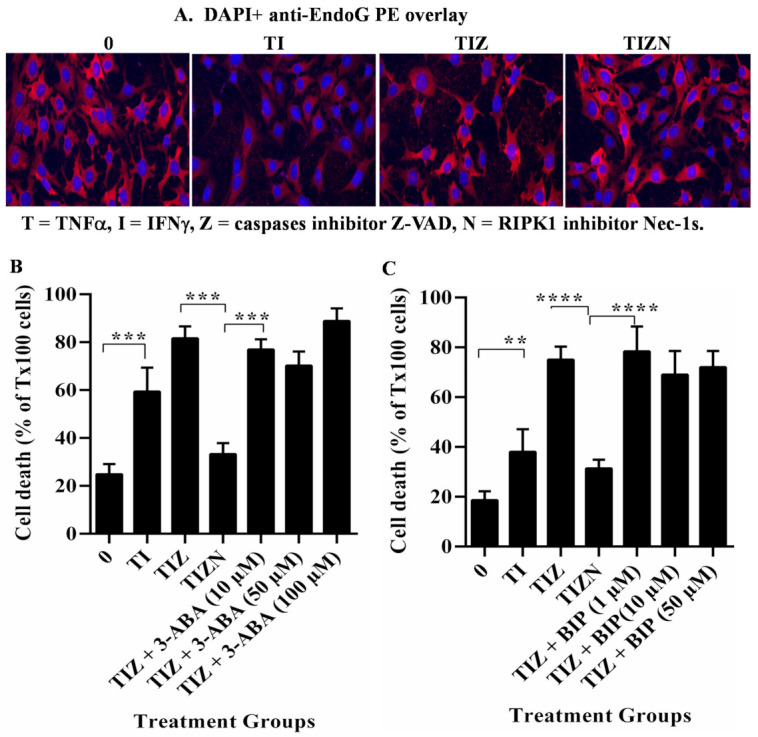
EndoG, PARP-1 and BAX do not participate in MVEC necroptosis. (**A**) WT MVECs were subjected to the in vitro simulation of IRI treatment as above and were fixed with 4% formaldehyde solution and permeabilized with 0.1% Triton X-100. The cells were incubated with antimurine EndoG followed by anti-rabbit PE-conjugated antibody. The nucleus was stained with DAPI. The same experiment was repeated three times. Representative individual cell images are shown. (**B**) MVECs were subjected to in vitro simulation of the IRI treatment. PARP-1 inhibitor 3-ABA or (**C**) BAX inhibitor BIP was added before the treatment. % EC death = sample count/count of Triton-X100-treated cells. Data are shown at 24 h post-reoxygenation as the mean ± SD of triplicates and representative of at least 3 independent experiments. ** *p* ≤ 0.01, *** *p* ≤ 0.001 and **** *p* ≤ 0.0001; 1-way ANOVA and Tukey’s multiple comparisons.

AIF itself does not have nuclease function but, rather, facilitates DNA cleavage via endonuclease G (EndoG) [45,46,47,48]. However, we did not observe the nuclear translocation of EndoG (Figure 5A). An early study demonstrated that the translocation of AIF requires the activation of poly(ADP-ribose) polymerase 1 (PARP-1) during cell death [49]. AIF translocation can be prevented by PARP inhibitors or the genetic deletion of PARP-1 [49]. However, in our study, MVEC necroptosis could not be inhibited with the addition of PARP-1inhibitor 3-aminobanzamide (3-ABA, Figure 5B). Other studies showed that Bcl-2-associated X (BAX) or the Bcl-2 Homology 3-interacting domain death agonist (BID) are required for AIF release [50,51,52]. However, in our study, adding BAX-inhibiting peptide V5 (BIP) did not prevent necroptosis (Figure 5C). These data imply that the function of AIF in necroptosis is associated with other molecule(s). 

### 2.4. CypD Deficiency in Donor Heart Graft Attenuates Graft IRI, AIF Translocation and Rejection

AIF not only contributes to nuclear damage but also participates in cell metabolic activities in the mitochondria. AIF deficiency causes mouse embryonic lethality [53,54]. Thus, a therapeutic strategy in vivo should consider blocking AIF release rather than gene deletion. Our in vitro data strongly supported the role of CypD in necroptosis under the in vitro simulation of IRI conditions. We wanted to examine if CypD deficiency in a donor graft can inhibit the translocation of AIF and prevent graft injury in mouse cardiac transplantation. 

**Figure 6:**
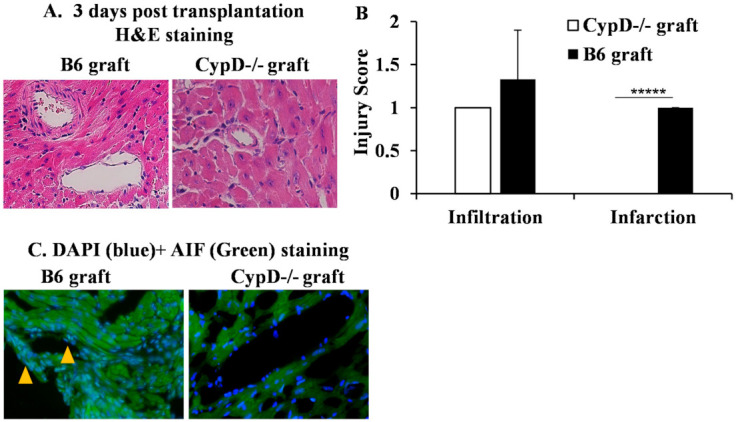
CypD deficiency in a donor graft attenuates IRI and AIF translocation. Cardiac grafts from wild-type and CypD^−/−^ B6 mice were subjected to ischemic storage at 4 °C for 4 h before being transplanted into BALB/c mice. Three days after, grafts were collected for H&E and AIF staining. (**A**) Pictures were taken at 200 times magnification and representative of 4 grafts in each group. (**B**) Graft injuries were scored in a blinded fashion and averaged from 4 grafts. ***** *p* ≤ 0.00001. Student’s *t*-test. (**C**) Graft sections were stained with anti-AIF-PE and DAPI. Pictures were taken at 200 times magnification. Colocalization of AIF and DAPI is indicated by arrows. Representative images are shown.

Firstly, we wanted to examine whether CypD deficiency in a donor graft could prevent IRI in cardiac transplant. Donor hearts from wild-type and CypD^−/−^ B6 mice were subjected to ischemic storage at 4 °C for 4 h and then transplanted into allogeneic BALB/c mice. Grafts (*n* = 4/group) were collected 3 days after transplantation for the pathology analysis. Double-blinded histological scores showed significant graft damage in the wild-type allografts compared with the CypD^−/−^ allografts (Figure 6A,B). Next, we analyzed AIF in the graft. Interestingly, the colocalization of AIF with the nucleus was found in the wild-type graft, not in the CypD^−/−^ graft (Figure 6C). Hence, these data indicate that CypD deficiency prevents graft IRI and the nuclear translocation of AIF.

**Figure 7:**
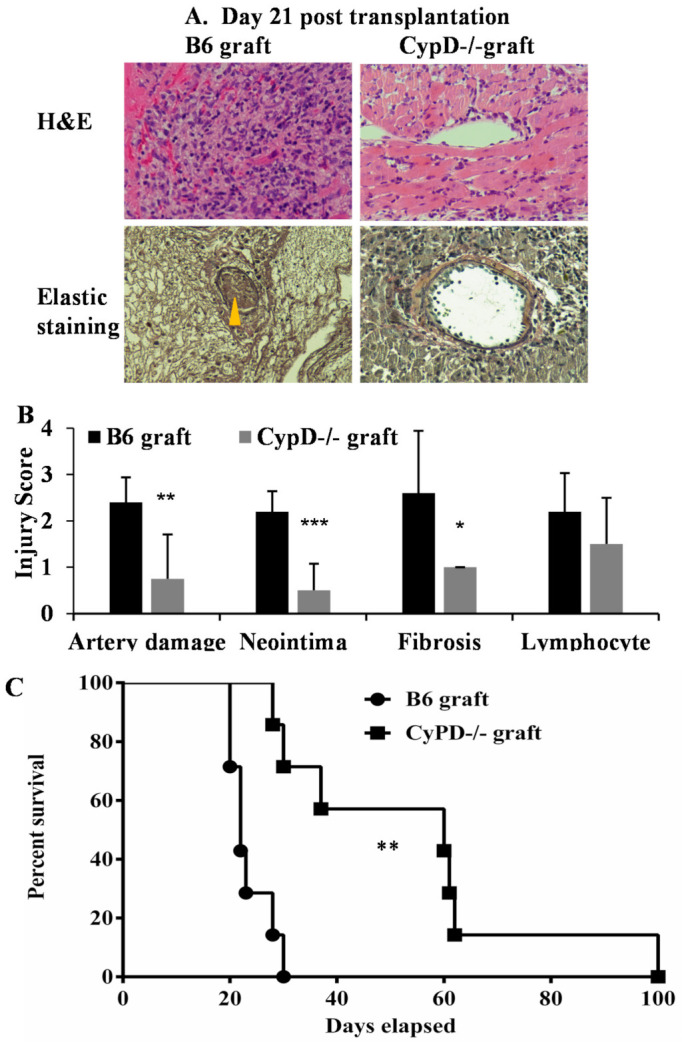
CypD deficiency in donor graft attenuates long-term graft injury and promotes post-transplant survival. (**A**) Cardiac grafts from wild-type B6 and CypD^−/−^ mice were treated with ischemic storage at 4 °C for 4 h and transplanted into BALB/c followed by rapamycin treatment. Recipient mice (*n* = 4/group) were euthanized 21 days post-transplantation, and the grafts were collected for H&E and elastic staining. Pictures were taken at 200 times magnification. Neointima is pointed to by the yellow arrow. Representative slide images are shown. (**B**) Graft injuries were quantified on a scale of 0-4, as above. * *p* ≤ 0.05, ** *p* ≤ 0.01 and *** *p* ≤ 0.001, 2-way ANOVA and Tukey’s multiple comparisons test. (**C**) Hearts from wild-type B6 and CypD^−/−^ mice were treated with ischemic storage at 4 °C for 4 h and transplanted into BALB/c, followed by rapamycin treatment. The cessation of cardiac beating is considered as rejection. *n* = 7 per group, ** *p* ≤ 0.01. Log-rank test.

To determine if CypD deficiency in donor cardiac grafts can attenuate a transplant injury long term, wild-type B6 or CypD^−/−^ hearts (*n* = 4/group) were subjected to ischemic storage as above and transplanted into BALB/c mice followed by a brief immunosuppression with sirolimus (rapamycin, days −1 to 9). The grafts were collected 21 days after the for pathological analysis. Double-blinded histological evaluation indicated significant damage to the microvasculature (Hematoxylin & Eosin (H&E) staining), with high levels of neointima formation and fibrosis (elastic stain) in the wild-type allografts (Figure 7A,B). Allograft injury was significantly decreased in the CypD^−/−^ allografts.

Finally, we tested if CypD deficiency in donor cardiac grafts can improve the transplant survival. Wild-type B6 or CypD^−/−^ hearts were subjected to ischemic storage and transplanted into BALB/c mice, followed by brief immunosuppression. The graft survival was monitored weekly. CypD deficiency in donor grafts attenuates rejection and prolonged graft survival compared with wild-type graft transplantation (mean survival = 54 ± 25.2 days, *n* = 7 versus 23.5 ± 3.9 days, *n* = 7, *p* = 0.002, Figure 7C). In summary, these data demonstrate that CypD deficiency in donor graft attenuated graft IRI and prolonged transplant survival.

## 3. Discussion

In transplant clinics, ischemic time correlates with delayed graft function and organ failure. IRI induces cell death and promotes inflammation. IRI is associated with various forms of cell death programs including apoptosis and necrosis. Apoptosis has been well-defined in IRI studies. However, the inhibition of apoptosis has not translated into clinical treatment. Recent studies have revealed that cells undergo necroptosis when apoptosis is inhibited. In our previous studies, we have found that RIPK1and RIPK3 contribute to TNFα-induced necroptosis in cardiac endothelial cells and that necroptosis plays an important role in cardiac transplant rejection [27,28,29,30]. However, the role of IRI in endothelial cell death and cardiac transplantation has not been studied. In this study, we found that MVECs undergo necroptosis under cold hypoxia and warm reoxygenation, in addition to proinflammatory cytokine treatment, simulating in vivo IRI during transplantation. MVEC necroptosis is regulated by CypD and the nuclear translocation of AIF. Interestingly, CypD deficiency in the donor heart graft inhibits AIF translocation, mitigating IRI and subsequent allograft rejection. Our studies indicate that CypD and AIF play significant roles in MVEC necroptosis following IRI. Targeting CypD-regulated mitochondrial permeability and the nuclear translocation of AIF may be a plausible approach in formulating therapeutic strategies aimed at reducing IRI and improving allograft viability and function in transplantation.

While the upstream pathways of necroptosis are well-established, the role of mitochondria and the downstream mechanisms involved remain controversial. In this study, inhibition of the mPTP molecule CypD attenuated necroptotic death under hypoxic conditions (Figure 1, Figure 2 and Figure 3). We were able to corroborate our previous finding that TNFα-induced necroptosis is regulated by CypD in a nonhypoxic model [29]. However, in this study, we observed AIF translocation to the nucleus under hypoxia-reoxygenation conditions, which is not seen during necroptosis under nonhypoxic conditions (data not shown). The mechanism regulating the processing and translocation of AIF to the nucleus is complex and not fully understood. The release of AIF from the mitochondria involves two essential steps: proteolytic cleavage yielding the truncated ‘liberated’ form of AIF and permeabilization of the mitochondria that enables its exit. Previous studies demonstrated that PARP-1, BAX or BID are required for AIF release [49,50,51,52]. However, in our study, adding either a PARP-1 inhibitor or a BAX inhibitor did not prevent necroptosis (Figure 5B,C). Our finding that the inhibition of CypD prevents cell death and AIF translocation to the nucleus suggests that CypD is a key factor for the release of AIF from mitochondria. However, it is still unclear how AIF is cleaved and liberated from mitochondrial membranes in our model. It is speculated that the cleavage of AIF to its liberated form under ischemia conditions is mediated by cysteine proteases such as calpains and cathepsins [55,56,57,58]. However, other studies indicated that calpain activation is not required for AIF translocation [59]. The mechanisms for calpain or cathepsin activation, cleavage and migration of AIF and the function of AIF in the nucleus in our model are still unknown, which are currently under investigation by our group. 

AIF and EndoG have been characterized as caspase-independent apoptosis inducers [45,46,47,48]. Recent studies suggested that AIF and EndoG play roles in necroptosis [60,61,62,63]. AIF itself does not have nuclease function and instead interacts with EndoG, which can cleave DNA. We observed the translocation of AIF (Figure 4). However, we did not observe the nuclear translocation of EndoG (Figure 5A), implying that other nuclease(s) mediate DNA degradation. A previous study showed that the macrophage migration inhibitory factor (MIF) has a nuclease function and is associated with AIF and PARP-1 in inducing DNA degradation [64]. However, the inhibition of PARP-1 did not prevent cell death in our study (Figure 5B). Therefore, it is still unknown if AIF interacts with other molecules in our system. 

We extended our in vitro findings to a murine IRI model of cardiac transplantation. CypD is a comprehensive target for long-term in vivo studies to prevent transplant injury, since CypD deficiency inhibits but does not eliminate mitochondrial permeability, and thus, normal mitochondrial function in the graft is conserved [65,66]. Previous studies showed that the inhibition of CypD showed a protective effect on cardiomyocyte apoptosis and reduced T-cell infiltration during acute cardiac graft rejection [67,68]. In our study, CypD deficiency in donor cardiac allografts reduced acute and chronic graft injury (Figure 6 and Figure 7A,B) and promoted heart transplant survival (Figure 7C). Interestingly, we have found that CypD deficiency limited AIF translocation in the graft (Figure 6C). Hence, our studies suggest that CypD plays an essential role in the necroptotic pathway via regulating AIF translocation under IRI conditions. 

Our study has limitations, as we only focused on necroptosis. IRI is a complex phenomenon involving multiple inflammatory responses and cell death mechanisms. Other death pathways such apoptosis or pyroptosis may contribute to cardiac graft injury and rejection. In future studies, we will consider investigating the effect of multiple death mechanisms on organ injury and transplant rejection. In our study, the ratio of AIF staining in the nucleus varies between experiments. It might be caused by some cells being lost after the fixing, permeabilizing, antibody binding and washing steps in immunocytochemistry. This requires further troubleshooting in future experiments. 

## 4. Materials and Methods

### 4.1. Mice

Wild-type C57BL/6 (B6), BALB/c and B6 CypD^−/−^ mice were purchased (Jackson Laboratory, Bar Harbor, ME, USA). The mice were maintained in the Animal Care and Veterinary Services facility at Western University.

### 4.2. Ethic Statement

All animal experimental procedures were approved by the Animal Care Committee (ACC) of Western University (Protocol 2019-131, approved by 1 February 2019 and is valid until 1 February 2024).

### 4.3. In Vitro Cold Hypoxia and Warm Reoxygenation 

MVECs from wild-type, CypD^−/−^ and RIPK3^−/−^ mice hearts were isolated and characterized as per the established protocol described previously [28,29]. Cells were seeded in triplicate in a 96-well plate in Dulbecco’s Modified Eagle’s Medium (DMEM; Thermo Fisher Scientific, Mississauga, ON, Canada) with 10% fetal bovine serum and 1% penicillin and streptomycin. 

To simulate the organ ischemia-reperfusion condition in vitro, cells were treated in deoxygenated serum and glucose-free DMEM under hypoxic conditions in anaerobic GENbags (BioMérieux, Montreal, QC, Canada) at 4 °C for 24 h before replacement with a normal cell culture medium with 1-μg/mL cycloheximide and cultured in a normoxic incubator at 37 °C with 20% O_2_ and 5% CO_2_. Cell death was detected and quantified by SYTOX Green (Thermo Fisher Scientific, Mississauga, ON, Canada) and the IncuCyte Live Cell Analysis System (Essen Bioscience, Ann Arbor, MI, USA). SYTOX Green is impermeable to live cells but crosses the compromised plasma membranes of dead cells and stains nucleic acids. 

Some (50 ng/mL) TNFα and IFNγ (PeproTech, Rocky Holl, NJ, USA) were added before hypoxia and reoxygenation to simulate the ischemia-reperfusion microenvironment in vivo. Pan-caspase inhibitor z-VAD-fmk (30 μM, ApexBio Technology, Houston, TX, USA) was added to inhibit apoptosis. RIPK1 was inhibited by the RIPK1 specific kinase inhibitor Nec-1s; (10 μM, Millipore Sigma, Etobicoke, ON, Canada). CypD was inhibited by CsA (Sigma-Aldrich, Oakville, ON, Canada) at 0.5–20 μM. FK-506 (Sigma-Aldrich), which is a calcineurin inhibitor but not a CypD inhibitor, was used as the control. The ROS scavengers used included the superoxide dismutase mimetic TEMPOL (Sigma-Aldrich) at 5–20 μM, the glutathione precursor NAC (Sigma-Aldrich) and the mitochondrion specific superoxide dismutase mimetic mito-TEMPOL (Sigma-Aldrich) at 5–20 μM. The PARP-1 inhibitor 3-ABA (Millipore Sigma) at 10–100 μM was used to inhibit PARP-1. BAX was inhibited by BIP (Millipore Sigma) at 1–50 μM.

### 4.4. RNA Interference 

MVECs were seeded on 6-well plates and grown to 60–80% confluency in normal cell culture medium. The cells were washed with PBS before transfection with AIF siRNA (ONTARGETplus, Dharmacon, Lafayette, CO, USA) using a transfection reagent (EndoFectin, Genecopoeia, Rockville, MD, USA). The siRNA-induced silencing of AIF expression was confirmed by PCR and Western blot, respectively, at 24, 48, 72 and 96 h post-transfection. Based on the PCR and Western blot results, the manufacturer-recommended dose of 200 nM was used for this study. The AIF-silenced cells were harvested at 48 h post-transfection for the cell death assays. Scrambled siRNA was used as the negative control (Thermo Fisher Scientific).

### 4.5. PCR

AIF expression was confirmed by real-time PCR. Total RNA from scrambled siRNA-transfected cells and AIF siRNA-transfected cells was extracted using TRIzol Reagent (Thermo Fisher Scientific). cDNA was generated from RNA using SuperScript™ II Reverse Transcriptase (Thermo Fisher Scientific). Real-time PCR was performed using QPCR Master Mix (Brilliant II SYBR^®^ Green, ABM, Vancouver, BC, Canada) and the Real-Time PCR System (Bio-Rad, Hercules, CA, USA). The *AIF* primers used were as follows: AIF forward primer (5′–3′) GTA GAT CAG GTT GGC CAG AAA CTC and reverse primer (5′–3′) GGA TTA AAG GCA TGT GCC AAC ACG. β-actin was used as an endogenous control for gene expression analysis. The β-actin primers used were as follows: β-actin forward primer (5′–3′) CCA GCC TTC CTT CCT GGG TA and reverse primer (5′–3′) CTA GAA CAT TTG CGG TGC A. The ΔCt values for AIF and β-actin were used to calculate the expression fold change.

### 4.6. Western Blots

The siRNA-induced silencing of AIF protein expression was confirmed by Western blot. The total protein from scrambled siRNA-transfected cells and AIF siRNA-transfected cells was extracted using RIPA Lysis and Extraction Buffer (Thermo Fisher Scientific) with protease inhibitors (Thermo Fisher Scientific). The concentration and purity of the isolated protein was measured by the Bradford Dye Protein Assay and spectrophotometer (Thermo Fisher Scientific). The protein samples were then equally loaded in the wells of a 10% SDS-PAGE gel. The separated protein samples were transferred onto a polyvinylidene fluoride (PVDF) membrane. AIF was detected by rabbit anti-mouse AIF antibody (Abcam, Toronto, ON, Canada) and HRP-conjugated goat anti-rabbit antibody (Cell Signaling Technology, Danvers, MA, USA), followed by a chemiluminescent substrate (Millipore Sigma) and imaging in a FluorChem M Imaging System (Protein-Samples, Ottawa, ON, Canada). Murine anti-mouse β-actin antibody (Millipore Sigma) and rabbit anti-mouse GAPDH antibody (Abcam) were used as the controls.

### 4.7. Immunocytochemistry

MVEC cultures were treated as described above. The cells were fixed with 4% paraformaldehyde and then permeabilized by 0.1% Triton X-100. The cells were incubated with primary rabbit anti-mouse AIF antibody or anti-EndoG and secondary donkey anti-rabbit PE-conjugated antibody (Abcam). Nuclei were detected by diamidino-2-phenylindole dihydrochloride (DAPI, Thermo Fisher Scientific). Images were acquired via fluorescent microscopy (Inverted ECLIPSE Ts2R, Nikon, Mississauga, ON, Canada).

### 4.8. DNA Analysis

DNA was extracted from MVECs using the phenol/chloroform method (Thermo Fisher Scientific) and quantified by a NanoDrop spectrophotometer (Thermo Fisher Scientific). DNA was analyzed on 0.8% agarose gel at 3 to 4 V/cm and stained with SYBR™ Safe DNA Gel Stain (Thermo Fisher Scientific) before being imaged by the FluorChem M Imaging System (Protein-Simple). Quick-Load 1-kb Extended DNA ladder (New England Biolabs, Whitby, ON, Canada) was used for DNA size reference. Methylnitronitrosoguanidine (MNNG, Sigma-Aldrich) was used as a positive control to induce DNA fragmentation. 

### 4.9. Cardiac Transplantation 

Donor hearts from WT and CypD^−/−^ mice were heterotopically transplanted into the abdominal region of BALB/c mice. The mice were anesthetized with a mixture of ketamine/xylazine before the transplantation procedures. Donor hearts were removed after clamping the aortae, vena cavae and pulmonary arteries and veins. They were then flushed with cold saline and stored in lactated Ringer’s buffer (Baxter, Deerfield, IL, USA) at 4 °C for 4 h before heterotopic transplantation into the abdominal cavity in recipient mice. Aortae from the donor hearts were sutured to the recipient mice abdominal aortae, and the donor heart pulmonary arteries were sutured to the recipient mice inferior vena cavae. The vena cavae and pulmonary veins in the donor hearts were sutured shut. The hearts were observed for spontaneous contractions following transplantation before the midline incisions of the recipient mice were sutured close. 

Following the transplantation procedure, the graft recipients received the immunosuppressant sirolimus (rapamycin, 1 mg/kg; LCL Laboratories, Woburn, MA, USA) from one day before the operation to postoperative day 9. Graft survival was monitored daily by abdominal palpation for pulse detection. The cessation of or significant drop in pulsation was considered as graft rejection and confirmed by a histopathological analysis.

### 4.10. Histology and Immunohistochemistry

Cardiac allografts in the recipient mice were collected and then flushed with normal saline, fixed with formalin and embedded in paraffin for sectioning. The tissue sections were then stained with H&E and elastic stain to evaluate the damage to the microvasculature, neointima formation and fibrosis by a pathologist in a blinded manner. The following changes were evaluated and quantified: microvasculature damage, neointima formation, fibrosis and leukocyte infiltration. The criteria used to score the injury on a scale of 0–4 were as follows—0: no change, 1: 0–24% change, 2: 25–49% change, 3: 50–74% change and 4: >75% change.

To detect AIF translocation to the nucleus from the mitochondria in MVECs, cell cultures were subjected to the in vitro cold hypoxia-reoxygenation injury model described earlier. Immunohistochemistry was performed according to the manufacturer’s protocols. The cells were incubated with rabbit anti-mouse AIF antibody followed by donkey anti-rabbit PE-conjugated antibody (Abcam). DAPI (Thermo Fisher Scientific) was used for nuclear counterstaining. Fluorescent images were acquired via microscopy (Nikon Inverted ECLIPSE Ts2R). 

### 4.11. Inclusion and Exclusion Criteria

Experiments that were repeated over 3 times were included in the conclusion and statistical analysis. All results from the experimental failures were excluded, including unexpected mouse death and in vitro experimental data without proper positive and negative controls. 

### 4.12. Statistical Analysis

Data was analyzed using the Student’s *t*-test or 1- and 2-way ANOVA with Tukey’s post-hoc corrections test. The Mantel–Cox log-rank test was used to determine the graft survival differences (Prism 4, GraphPad Software, San Diego, CA, USA. Differences were considered significant when the *p*-value ≤ 0.05.

## 5. Conclusions

Our in vitro and in vivo studies confirmed that necroptosis plays a significant role in IRI-induced endothelial cell death and transplant damage. CypD regulates the nuclear translocation of AIF and necroptosis and, thus, is an effective target to prevent IRI and transplant rejection.

## Data Availability

The data presented in this study are available on request from the corresponding author.

## Abbreviations

3-ABA	3-aminobanzamide
BAX	Bcl-2-associated X.
BID	Bcl-2 Homology 3-interacting domain death agonist.
CsA	Cyclosporin A.
CypD	Cyclophilin D.
DAPI	Diamidino-2-phenylindole dihydrochloride
MVEC	mouse microvascular endothelial cells.
NAC	Nacetyl-L-cysteine.
Nec-1s	Necrostatin-1s.
PARP-1	poly(ADP-ribose) polymerase 1
PCR	polymerase chain reaction.
RIPK	receptor interacting protein kinase.
ROS	reactive oxygen species.
TEMPOL	tetramethylpiperidine-oxyl.
Z-VAD-fmk	Z-Val-Ala-Asp-fluoromethylketoe.
